# Nature Experiences and Adults’ Self-Reported Pro-environmental Behaviors: The Role of Connectedness to Nature and Childhood Nature Experiences

**DOI:** 10.3389/fpsyg.2018.01055

**Published:** 2018-06-26

**Authors:** Claudio D. Rosa, Christiana Cabicieri Profice, Silvia Collado

**Affiliations:** ^1^Regional Development and Environment, Universidade Estadual de Santa Cruz, Ilhéus, Brazil; ^2^Department of Psychology and Sociology, Universidad de Zaragoza, Teruel, Spain

**Keywords:** connection to nature, child, ecological behavior, leisure, nature exposure, outdoor recreation, path analysis

## Abstract

This cross-sectional study aims to improve our understanding of the psychological pathways behind the commonly reported link between experiences in nature and pro-environmentalism. Particularly, we explore whether nature experiences lead to self-reported pro-environmental behaviors (PEBs) and whether this relation is mediated by connectedness to nature. Additionally, we examine the possible lasting effect of childhood experiences with nature on adults’ PEB. Most studies reporting on the link between contact with nature and pro-environmentalism have been conducted in developed countries, limiting the generalization of the results. To address this gap in the literature, the current study was conducted in a developing country (Brazil) with a sample of 224 young adults. According to our findings, greater contact with nature during childhood is associated with greater contact with nature as an adult, which, in turn, is positively associated with connectedness to nature and PEB. The stimulation of pleasant experiences while in direct contact with nature during childhood seems to trigger interactions with nature in adulthood and consequently, adults embrace pro-environmental actions.

## Introduction

It is increasingly evident that environmental issues such as the loss of biodiversity and climate change are caused by human action (Steffen et al., 2015) and that behaving in a non-pro-environmental way can have severe consequences both for the planet and its inhabitants (Schultz and Kaiser, 2012). Consequently, one would expect people to exhibit behavior that leads to a more environment-friendly lifestyle. Environmental psychologists have long investigated different strategies and pathways to enhance pro-environmental behavior (PEB) (Winkel et al., 2009). For example, research that focuses on social norms has suggested that seeing another person conducting a pro-environmental action enhances the observer’s probability of engaging in the same behavior (Cialdini and Trost, 1998). Further research has included a focus on the values and attitudes leading to PEB (Steg and Vlek, 2009), information campaigns (McKenzie-Mohr, 2008), and environmental education (Zelezny, 1999).

A new and innovative line of research about the promotion of PEB draws on the hypothesis that direct contact with nature is positively associated with pro-environmental attitudes and PEB (Larson et al., 2011a; Chawla and Derr, 2012; Cheng and Monroe, 2012; Collado et al., 2015; Otto and Pensini, 2017; Whitburn et al., in press). In line with this, it has been suggested that reducing opportunities to have contact with nature can lead to an amplified feeling of human-nature dissociation (Chawla and Derr, 2012), which may, in turn, hinder support for environmental causes (Soga and Gaston, 2016).

Positive experiences in nature, as reported by adults (Wells and Lekies, 2006; Hinds and Sparks, 2008; Larson et al., 2011b) and children (Larson et al., 2011a; Collado et al., 2015), have been positively associated with higher engagement on PEB. We define positive experiences in nature as those experiences in which the individual can engage freely with the environment (Chawla and Derr, 2012). For example, previous studies found a positive association between adults’ recreational contact with nature (e.g., birdwatching, camping, and fishing) and PEB (Nord et al., 1998; Cooper et al., 2015). Similarly, exposure to nature (e.g., camping outdoors) is associated with greater connectedness to nature and PEB (Pensini et al., 2016). Soga et al. (2016) demonstrated that 9 to 12 year-olds’ direct experiences with nature (e.g., picking plants or flowers) were linked to their willingness to conserve biodiversity.

Despite the extensive and robust research literature identifying the link between positive experiences in nature and PEB (Chawla and Derr, 2012), the processes behind this relation are still unclear (Wells and Lekies, 2006; Schultz and Kaiser, 2012; Whitburn et al., in press). Explanations have been offered to understand the relation between experiences in nature and PEB, such as psychological restoration (Byrka et al., 2010) and environmental attitudes (Wells and Lekies, 2006; Collado et al., 2015). The aim of the present study is to extend an understanding of the mechanisms that connect exposure to nature to PEB, by exploring the possible explanatory role of connectedness to nature. We also consider childhood contact with nature as an important factor leading to adults’ PEB (Chawla and Derr, 2012; Collado et al., 2015; Otto and Pensini, 2017; Evans et al., 2018).

Connectedness to nature can be defined as a trait related to the feeling of emotional connection with the natural environment (Mayer and Frantz, 2004). As far as we know, only three studies considered the possible mediating role of connectedness to nature in the relation between exposure to nature and PEB. Pensini et al. (2016) found a direct link between exposure to nature and PEB. The authors used a composite measure of direct (e.g., camping outdoors) and indirect (e.g., looking at the stars) contact with nature. The effect of contact with nature on PEB was partially explained by connectedness to nature. Unfortunately, the mediational role of connectedness to nature was analyzed using only regression analyses and the authors did not provide fit indices. Similarly, Otto and Pensini (2017) found that children’s connectedness to nature partially mediated the relation between visits to nature-based environmental educational facilities or programs and ecological behaviors. Due to the study design, it is difficult to distinguish between the impact of exposure to nature and the impact of environmental education on children’s increased connectedness to nature and ecological behaviors. More recently, Whitburn et al. (in press) have used structural equation modeling to evaluate the mediating effect of connectedness to nature on the relation between exposure to nature and PEB. Exposure to nature was recognized as the percentage of neighborhood vegetation cover. In contrast to Pensini et al. (2016), exposure to nature within the neighborhood did not promote connectedness to nature.

Given the limited number of studies that consider connectedness to nature as a psychological pathway behind the relation between contact with nature and PEB and the mixed results found, the current study extends previous research in three ways. First, we focus on connectedness to nature as a possible mediator between adults’ positive experiences in nature and PEB with a sample from a developing country. This will be, to the best of our knowledge, the first time this connection has been researched. Given that the way people relate to nature varies across cultures (Milfont and Schultz, 2016), and that different experiences with nature have a distinct impact on people’s connectedness to nature (Giusti et al., 2018), it seems relevant to include developing countries when examining the relation between humans and nature. This will allow us to identify whether the relations between nature experiences, connectedness to nature, and PEB found in previous studies hold true for people in developing countries, and to widen our understanding of the transaction between people and environments in different cultures (Clayton et al., 2016). Second, studies evaluating the mediating role of connectedness to nature in the relation between exposure to nature and PEB have recognized contact with nature as the amount of vegetation within the neighborhood (Whitburn et al., in press), visits to educational nature facilities (Otto and Pensini, 2017), and general contact with nature both directly and indirectly. This broad recognition of contact makes it difficult to examine the effect of direct experiences in nature, as other variables such as indirect contact with nature and education may influence the results. In the current study, we investigate the relation between positive exposure to nature, connectedness to nature, and PEB using a measure of reported direct contact with nature. Third, childhood experiences in nature have been identified as an important factor influencing adults’ contact with nature (Thompson et al., 2008). Therefore, we examine whether the effects of childhood contact with nature on PEB described in previous studies (Wells and Lekies, 2006; Larson et al., 2011b; Pensini et al., 2016) can be explained by adults’ current contact with nature. In the next sections, we review previous literature on the link between nature experiences and pro-environmentalism and set up the bases for the current study.

### Experiences in Nature and Pro-environmentalism

Research has shown that direct contact with nature is related to several positive outcomes; including, uplifted mood (Joye and Bolderdijk, 2015), revitalization (Ryan et al., 2010), and psychological restoration (Hartig, 2004; Staats, 2012; Carrus et al., 2017). Exposure to nature also seems to increase pro-environmentalism. For example, in an early study, Dunlap and Heffernan (1975) found a correlation between outdoor recreation and environmental concern. In line with these results, Lawrence (2012) reported that undergraduate students who visited natural areas as a requirement of their course had a stronger identification with the place and reported greater engagement with responsible environmental behaviors (e.g., joining community clean-up efforts). Similarly, Bjerke et al. (2006) observed an association between preference for outdoor recreation activities and positive environmental attitudes. Similarly, research with children suggests those who reported having more frequent experiences with wild animals and plants also reported greater biophilia and willingness to conserve animals (Zhang et al., 2014).

Despite much evidence supporting the relation between the exposure to nature and pro-environmentalism (Collado et al., 2015; Crawford et al., 2017), the strength of this relation differs between studies (Chawla and Derr, 2012). One reason for this may be that the independent (direct and/or indirect contact with nature) and dependent (PEB, environmental attitudes, concern, and knowledge) variables differ across studies. As a result, it is unclear which factors and processes have the most significant impact on the relation between contact with nature and pro-environmentalism. Therefore, it is important to study the effects of positive experiences in nature on people’s pro-environmentalism.

Both empirical evidence and theory support the notion that experiences in nature can promote connectedness to nature (Mayer and Frantz, 2004; Barton et al., 2016; Crawford et al., 2017; Richardson and Sheffield, 2017; Giusti et al., 2018). Furthermore, connectedness to nature seems to favor PEB engagement (Mayer and Frantz, 2004; Tam, 2013; Whitburn et al., in press). For example, children who experienced nature in parks reported feeling a greater connection to nature and claimed that the visit made them want to take better care of the local environment (Crawford et al., 2017). In a study with adults, present and past experiences in nature explained 39% of emotional affinity toward nature’s variance and 43% of interest in nature topics variance. These, in turn, explained reported PEB (Kals et al., 1999). Despite the existence of other possible mediators in the relation between nature experience and PEB, connectedness to nature has been suggested as one of the most prominent ones (Kals et al., 1999; Collado et al., 2013; Pensini et al., 2016; Whitburn et al., in press); however, its role has been scarcely considered (Pensini et al., 2016; Otto and Pensini, 2017; Whitburn et al., in press). The effect of childhood experiences in nature on adults’ PEB while considering connectedness to nature is also under-researched.

There is general agreement that childhood experiences in nature have a lasting effect on adults’ pro-environmentalism (Wells and Lekies, 2006; Chawla and Derr, 2012; Evans et al., 2018). Previous studies have demonstrated that people who participated in outdoor leisure activities as a child tend to engage in these activities as an adult (Bixler et al., 2002; Thompson et al., 2008). Studies with adults have found a positive association between reported contact with nature during childhood, attitudes, and PEB (Ewert et al., 2005; Wells and Lekies, 2006). However, we found just two studies that analyzed this relation while also considering adults’ current contact with nature (Larson et al., 2011b; Pensini et al., 2016). Both studies showed that for people living in developed countries, nature experiences during childhood have an indirect effect on PEB through current nature experiences. Larson et al. (2011b) demonstrated, with a sample of 319 adult state park visitors, that contact with nature during childhood had an indirect relation with biocentric values and PEB through current experiences with nature. Similarly, Pensini et al. (2016) concluded that nature exposure during childhood had an indirect effect on connectedness to nature and ecological behavior through current nature exposure. After controlling for current nature exposure, experiences in nature during childhood had no significant correlation with connectedness to nature and ecological behavior (Pensini et al., 2016). The authors did not provide fit indices of their results, so it is not possible to assess how adequately these relations represent their data.

### The Present Study

The findings from the aforementioned studies suggest that contact with nature during childhood has a positive effect on adults’ PEB through their current contact with nature. Our aim is to evaluate if and how current and past experiences in nature relate to PEB while considering connectedness to nature. Thus, we analyze the possible mediating effect of connectedness to nature on the relation between current experiences in nature and PEB, and the possible mediating effect of current experiences in nature on the relation between childhood experience in nature, connectedness to nature, and PEB. We use path analysis as a more robust statistical technique than regression analysis (Byrne, 2010). Furthermore, rather than using a combination of direct (e.g., camping outdoors) and indirect (e.g., looking at the stars) experiences in nature (Pensini et al., 2016), we focus on the unique effect that direct positive experiences in nature may have on PEB. By extending the understanding in previous studies that focus on developed countries, we focus on a sample of adults from a developing country: Brazil.

It has been stated previously that the way individuals relate to and interact with nature differs across cultures (Milfont and Schultz, 2016). Whiting et al. (2017) found that the main motivation for Latinos to visit state parks were social interactions, whereas non-Hispanic White visitors tend to give less prominence to social factors. Thus, different ethnic groups may have distinct motivations to interact with nature, and it is therefore more likely that they have varied outcomes as a result of these experiences (e.g., socialization, restoration, fitness). In line with this idea, the Brazilians’ view of the relation between humans and nature may differ from those of individuals in developed countries (Bechtel et al., 1999; Vikan et al., 2007). Bechtel et al. (1999) argue that North Americans see nature preservation and economic growth as irreconcilable; whereas, Brazilians believe economic prosperity does not necessarily imply nature degradation. However, the relation between past and current experiences in nature, connectedness to nature, and PEB has not, to the best of our knowledge, been researched in a developing country. Thus, we base our hypotheses on theoretical background and previous studies in this area in developed countries while keeping an open mind for possible differences that may appear given the distinct interaction people in developed/developing countries have with nature.

**FIGURE 1:**
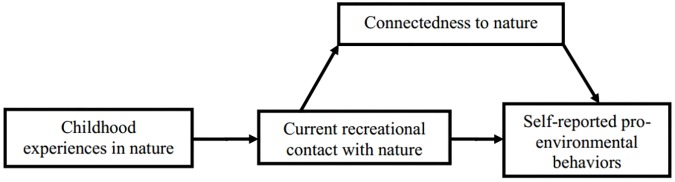
Hypothesized relationships between constructs.

Based on previous studies (Pensini et al., 2016), we expect the association between adults’ current experiences in nature and PEB to be partly explained by connectedness to nature (Hypothesis 1). We also examine whether the relation between childhood experience in nature, connectedness to nature, and PEB is mediated by current experiences in nature. Empirical evidence supports the notion that lasting effect of childhood experiences in nature on adults’ pro-environmentalism is principally indirect—through adults’ current experiences in nature (Larson et al., 2011b; Pensini et al., 2016). Considering this, we hypothesize that reported positive experiences in nature during childhood will have a direct effect on adults’ current contact with nature, and through it, will be associated with connectedness to nature and PEB (Hypothesis 2) (see **Figure 1**). Our data are correlational, so causality claims should be considered with caution, as they are based on correlations and theory.

## Materials and Methods

### Participants and Place of Study

Participants were 224 undergraduate students (140 women and 84 men) from a University in northeastern Brazil. They were enrolled in different courses including pedagogy, biology, law, physical education, and economics. The mean age was 23.64 years old (*SD* = 5.96). Students were selected in this study because young adults tend to be more active than older adults and, consequently, have more contact with nature (Hallal et al., 2012). Additionally, the region where the study took place is famous for its natural beauty (e.g., beaches, green urban areas, and conservation unities) and opportunities for contact with nature (e.g., swimming at beach, visiting green parks, camping, hiking, and fishing).

### Measures

The following measures were used:

*(a) Positive contact with nature during childhood:* Following Larson et al.’s (2011b) approach, an item was used to register participants’ direct contact with nature during childhood. This was, “How frequently did you participate in leisure activities in contact with nature during childhood. Leisure activities in nature include visiting natural places, playing soccer or volleyball at beach, swimming, surfing, camping, hiking, etc.” Respondents rated on a scale from 1 = never to 5 = most of the days.

*(b) Current positive contact with nature:* This was recorded using an item similar to that used by Larson et al. (2011b). This was, “How frequently do you participate in leisure activities in contact with nature. Leisure activities in nature include visiting natural places, playing soccer or volleyball at beach, swimming, surfing, camping, hiking etc.” Similar to the previous measure, participants could rate their response on a scale of 1 = never to 5 = most of the days.

*(c) Connectedness to nature:* This was registered with the Connectedness to Nature Scale (Mayer and Frantz, 2004). We used the Brazilian version of this scale consisting of 13 items that participants rated on a 5-point Likert scale: 1 = strongly disagree to 5 = strongly agree (Pessoa et al., 2016). The unidimensional structure of this scale has already been confirmed with Brazilians (Pessoa et al., 2016). Cronbach’s alpha in the present study was 0.83.

*(d) Self-reported PEB:* This was identified using a self-reported PEB scale employed by Larson et al. (2015). It was chosen for the following reasons: (1) The authors developed the scale from previous research on PEB. (2) The authors conducted several interviews in order to identify behaviors people perceive as beneficial for local environmental quality. (3) The authors included behaviors that required various levels of effort to participate in, which included daily conservation actions (e.g., saving energy at home) and environmental citizenship behaviors (e.g., donating money to support environmental protection). This approach allowed for greater variance in participant response. (4) It has been demonstrated that the scale captures the effect of recreational contact with nature in self-reported PEB (Cooper et al., 2015). The original scale consists of 13 items and four dimensions: conservation lifestyle, land stewardship, social environmentalism, and environmental citizenship. In the current study, the stewardship dimension (3 items) was removed as it included behaviors that participants in this sample would not normally engage in (e.g., made my yard or my land more desirable for wildlife). The remaining 10 items were translated following a back translation procedure with the collaboration of two faculty members of a postgraduate program. One faculty member translated the items from English to Portuguese, and the other, in possession of that version, translated them back to English. Substantial variations were not identified. An exploratory principal axes analysis with oblique (oblimim) rotation was conducted (Kaiser–Meyer–Olkin [KMO] = 0.807 and Bartlett’s Test of Sphericity χ^2^_(45)_ = 678.738, *p* < 0.001). Three eigenvalues greater than one were found (Table 1 in Supplementary Material). Following Henson and Roberts (2006), the pattern and structure matrices were interpreted. The pattern matrix shows that six items load more strongly on the first factor, two items on the second, and two on the third. However, we do not consider the second and third two-item factors separately for two reasons: factors formed by less than three items are not advisable (Fabrigar et al., 1999) and these two factors did not match Larson et al.’s (2015) proposed factorial structure. Moreover, the structure matrix shows that all items correlate at least moderately with the first factor (Table 2 in Supplementary Material). We used parallel analysis (PA) (1,000 replications) to determine the number of factors to be retained (Fabrigar et al., 1999; Damásio, 2012). Considering results from PA, factor loadings, and internal consistency (α ≥ 0.80), a unidimensional structure was deemed the most adequate for the PEB scale in the present study (Fabrigar et al., 1999; Damásio, 2012). This unidimensional structure was checked through a confirmatory factor analysis and was deemed acceptable: χ^2^_(34)_ = 91.626, χ^2^/*df* = 2.70 (*N* = 216, *p*< 0.001), CFI = 0.91, AGFI = 0.87, GFI = 0.92, and RMSEA = 0.09. All items were reported on a scale from 1 = never to 5 = very often. Cronbach’s alpha was 0.82.

The mean scores of each scale were used to represent the constructs.

### Procedure

Participation was anonymous and voluntary. Participants answered an online questionnaire that favors anonymity and reliable completion (Leeuw, 2008) and does not change the factorial structure of the instruments (Campos et al., 2011). The average time to complete the questionnaire was 15 min.

### Data Analysis

Responses from two participants were removed from the dataset, as they presented missing values in the contact-with-nature variables. Missing values (0.45%) were imputed using the mean value of each variable (Tabachnick and Fidell, 1996); responses from a further six participants were discarded because they were deemed multivariate outliers based on Mahalanobis distance (*p* < 0.001) (Tabachnick and Fidell, 1996). Following Harrington’s (2009) approach, univariate extreme cases were recoded to retain the highest scores but reduce the extremes. This data is available as Supplementary Material.

Descriptive and correlational analyses were conducted before moving to address the main hypotheses of the study. A path analysis was conducted with AMOS with 5000 resamples using bootstrapping (95% CI) (Hayes, 2009). We tested whether connectedness to nature partially explained the association between current recreational contact with nature and PEB (H1), and whether current recreational contact with nature fully explained the relation between nature experiences during childhood and adults’ connectedness to nature and PEB (H2) (see **Figure 1**). Contact with nature during childhood acted as an exogenous variable. Current contact with nature, connectedness to nature, and PEB acted as endogenous variables. The fit indexes used were χ^2^/*df* < 4, GFI > 0.90, CFI > 0.90, RMSEA < 0.08 (Harrington, 2009; Byrne, 2010). The indirect effects among the variables were also checked. The size of the indirect effect in at least 95% (confident intervals with a significant level α = 0.05) of the resamples should be either above or below 0 in order to conclude that there is a significant indirect effect.

## Results

Descriptive statistics and correlations between the variables are presented in **Tables 1, 2**, respectively. Participants report a medium–high feeling of connection to nature. However, participants seem to do little to conserve the environment. Additionally, participants recall spending quite a lot of time in nature as a child while nature experiences appear to be less frequent during young adulthood. Contact with nature during childhood and current contact with nature were positively associated with each other. Additionally, they were positively related to connectedness to nature and PEB. PEB and connectedness to nature were also positively associated.

**Table 1:** Mean values (standard deviation) of connectedness to nature (CN), reported pro-environmental behaviors (PEB), and contact with nature during childhood and currently (childhood CN and current CN).

	CN	PEB	Childhood CN	Current CN
Women	3.89 (0.51)	2.27 (0.63)	4.35 (0.75)	2.99 (1.04)
Men	3.73 (0.52)	2.23 (0.65)	4.25 (0.83)	3.04 (0.91)
Total	3.83 (0.52)	2.25 (0.64)	4.31 (0.78)	3.01 (0.99)

**Table 2:** Correlations among the variables.

	1	2	3	4
(1) Pro-environmental behaviors				
(2) Connectedness to nature	0.44^∗∗^			
(3) Contact with nature during childhood	0.19^∗∗^	0.15^∗^		
(4) Current contact with nature	0.43^∗∗^	0.42^∗∗^	0.27^∗∗^	

*^∗^p < 0.05; ^∗∗^*p* < 0.01*.

**FIGURE 2:**
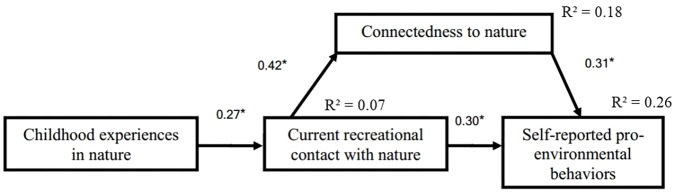
Results for the path analysis. All regression coefficients are standardized. ^∗^*p* < 0.001.

To examine the relation between positive experiences in nature and PEB, we conducted the path analysis depicted in **Figure 1**. The model fits the observed data well: χ^2^_(2)_ = 1.528, χ^2^/df = 0.76 (*N* = 216, *p* = 0.466), CFI = 1.00, AGFI = 0.982, GFI = 1.00, and RMSEA = 0.00; modifications indices did not suggest any changes. It explains 26% of PEB variance, of which 19% is explained by connectedness to nature and 7% by current recreational contact with nature. Additionally, 7% of the variance of current recreational contact with nature is explained by childhood experiences in nature, and 18% of the variance in connectedness to nature is explained by current recreational contact with nature (**Figure 2**). Analyses were also conducted without substituting the missing values and deleting or recoding outliers, and the results remained the same. All the relations remained statistically significant, in the same direction, and with similar strength.

The effect of current contact with nature on PEB was partially explained by connectedness to nature (β_indirect_ = 0.13, 95% CI = [0.07, 0.21]). Contact with nature during childhood had an indirect effect on connectedness to nature and PEB—fully explained by current contact with nature. Specifically, the indirect effect of childhood contact with nature on connectedness to nature through current experiences in nature was β = 0.12, 95% CI [0.06, 0.18]. The total indirect effect of childhood contact with nature on PEB through current contact with nature was β = 0.12, 95% CI [0.06, 0.18] (**Figure 2**). These results indicate a complementary mediation of connectedness to nature in the relation between current recreational contact with nature and self-reported PEB (Zhao et al., 2010; **Figure 2**). They also show an indirect-only mediation of current recreational contact with nature in the relation between childhood experiences in nature, connectedness to nature, and self-reported PEB. Direct effects are shown in **Figure 2**.

## Discussion

Extant evidence supports the link between positive experiences in nature and pro-environmentalism (Evans et al., 2007; Hinds and Sparks, 2008; Chawla and Derr, 2012; Collado et al., 2015). However, little is known about the psychological processes behind this link. Our study constitutes a first step toward clarifying the pathways behind the relation between contact with nature and pro-environmentalism in a developing country.

Our findings suggest that adults’ current experiences in nature have a positive effect on their PEB. In line with Pensini et al.’s (2016) findings, this effect is partially explained by connectedness to nature, supporting our Hypothesis 1. Contrary to Whitburn et al.’s (in press) results, exposure to nature was found to be associated with greater connectedness to nature (Kals et al., 1999; Mayer and Frantz, 2004; Pensini et al., 2016). It may be that an objective measure of exposure to nature, such as neighborhood vegetation cover, as used by Whitburn et al. (in press), does not account for possible differences in people’s experiences in nature (Giusti et al., 2018). In line with this, Whitburn et al. (in press) found that tree planting participation was significantly associated with greater connectedness to nature. Thus, living in a greener neighborhood alone does not ensure positive experiences in nature that will, in turn, improve connectedness to nature.

According to our results, connectedness to nature partially explains the association between current positive contact and PEB. While connectedness to nature has been suggested as one of the main predictors of PEB (Whitburn et al., in press), other possible mediators might help us understand the mechanisms behind the relation between positive experiences in nature and pro-environmentalism. For instance, psychological restoration (Collado and Corraliza, 2015), cognitive interest in nature (Kals et al., 1999), place identity (Lawrence, 2012), biocentric values (Larson et al., 2011b), and environmental beliefs (Collado et al., 2013) have also been suggested as possible mediators of the relation between nature experience and PEB. In line with this, Whitburn et al. (in press) found that exposure to nature promotes psychological restoration, which, in turn, promotes environmental attitudes and PEB. One could also consider that nature experiences may change our emotional identification with the setting where these experiences take place. This emotional identification could lead to a personal investment in the setting (Lawrence, 2012). Considering children, contact with nature has been found to be associated with greater affective attitudes toward biodiversity and willingness to conserve it (Soga et al., 2016). Though they are beyond the scope of this paper, these possible mediating paths await future research.

Quantitative and qualitative studies have indicated the importance of experiences in nature during childhood for the development of pro-environmental attitudes and PEB (Tanner, 1980; Palmer, 1993; Kals et al., 1999; Wells and Lekies, 2006; Soga et al., 2016). However, it is difficult to distinguish whether the changes that occur during childhood remain in adult life. In line with Hypothesis 2 and previous studies (Larson et al., 2011b; Pensini et al., 2016), after controlling for current contact with nature, contact with nature during childhood only had indirect effects in connectedness to nature and PEB. Thus, in accordance with previous research, it appears that the main effect of childhood experiences in nature is to stimulate the continuance of nature experiences later in life (Bixler et al., 2002; Thompson et al., 2008; Larson et al., 2011b; Pensini et al., 2016). This argument is supported by a longitudinal study that found children’s environmental attitudes at 6 years old do not predict their environmental behaviors 12 years later. When controlled for the variables investigated (e.g., child environmental behavior), the only predictors of young adults’ PEBs was time spent outdoors during childhood and maternal education (Evans et al., 2018). These results suggest that changes in pro-environmental attitudes in early life tended not to persist into young adulthood. Unfortunately, the authors did not control for the current time that participants spent outdoors. Based on our findings, it seems plausible that participants’ current contact with nature explains the link found between time spent outdoor during childhood and current PEBs.

Our study sheds light on the processes that lead childhood experiences in nature to pro-environmentalism later in life. To the best of our knowledge, this is one of the very few studies in which adults’ self-reported PEBs can be traced back to their childhood positive experiences in natural areas, and it is the first one to sample participants from a developing country. Even though differences have been found in the way people relate to nature in developed and developing countries (Gifford and Nilsson, 2014; Milfont and Schultz, 2016), our results are in concordance with previous findings in developed countries (Larson et al., 2011b; Pensini et al., 2016; Whitburn et al., in press). This suggests that differences in people’s relation with nature across cultures (Bechtel et al., 1999; Whiting et al., 2017) may not greatly influence the way people connect with nature. The development of connectedness to nature via positive nature experiences and the relation between connectedness to nature and PEB may not be influenced by the different cultural interactions with nature that people have (Milfont and Schultz, 2016; Whiting et al., 2017).

Participants’ infrequently reported acting in a pro-environmentally friendly way. We can only speculate why this may be the case. One reason may be the scale chosen to register PEB includes behaviors unlikely to be conducted by the general population. Although this is a strength of the scale in terms of participants’ response variance, it is more difficult to find people who actively engage in the most difficult behaviors. For example, voting to support pro-environmental policies depends upon annual election cycles (Larson et al., 2015). Another explanation may come from the fact that our participants had a medium frequency of contact with nature compared to those in previous studies (Larson et al., 2015). This seems to be due to the fact that Larson et al.’s (2015) sample was formed of birdwatchers, hunters, and landowners, whose contacts with nature are most likely higher than that of the general population. Recruiting participants from specific groups with higher contact with nature in Brazil may similarly result in higher PEB. Future studies considering different frequencies of contact with nature are certainly required. For example, cross-cultural studies could help us understand whether people’s contact with nature during childhood and adulthood varies according to culture, and how this might, in turn, affect their connectedness to nature and PEB.

Furthermore, in line with previous studies (Nord et al., 1998; Cooper et al., 2015; Evans et al., 2018), contact with nature does not explain a large amount of PEB variance. It suggests that other factors could also be influencing PEB, such as environmental education (Lankenau, 2018) and social and moral norms (Bamberg and Möser, 2007). Moreover, as more educated people tend to present lower response variance than less educated people, correlations tend to be small in university students’ samples in comparison to the general population (Meisenberg and Williams, 2008).

Some limitations should be considered when interpreting our results. First, our sample is non-probabilistic, and our design is cross-sectional, which hinders the generalization of results and causality inferences. It is also difficult to confirm that it is nature experiences that promote pro-environmentalism and not the other way around (Thapa, 2010). Nevertheless, given that the same pattern has been seen with adults (Richardson and Sheffield, 2017) and children (Collado et al., 2013), as well as the fact that we tested the opposite model and the statistics did not fit, we are fairly confident that nature experiences promote pro-environmentalism.

Second, the development of scales to measure both childhood and current positive contact with nature could overcome our limitation of using single-item measures. It is noteworthy that single-item measures are not necessarily inferior to multiple-item measures (Gardner et al., 1998; Brügger et al., 2011). However, a multiple-item measure would permit researchers to evaluate the internal consistency of the measure and the dimensions that may occur when recording people’s contact with nature. The design of measures to register different types of nature experiences, direct and indirect, recreational and compulsory, is also encouraged. This will enable researches to evaluate the possible differences in the effect various types of nature experiences have on pro-environmentalism.

Third, we cannot rule out the possibility that adults who perceive themselves as more pro-environmental overestimate their positive experiences in nature as children (Chawla and Derr, 2012). Future studies could also re-examine our findings with a sample more representative of the general population. Moreover, longitudinal investigations may help confirm the causal inferences and mitigate the recall limitation of this and previous studies (Wells and Lekies, 2006; Thompson et al., 2008).

Limitations aside, our findings suggest that children’s positive experiences in nature increase their likelihood of experiencing nature later in life and this, in turn, leads to pro-environmentalism. These results align with previous findings indicating that nature experiences are associated with pro-environmentalism (Soga and Gaston, 2016). Hence, children’s free contact with nature should be encouraged. This could be facilitated through environmental education programs aimed at increasing parental awareness about the benefits of contact with nature for their children. Contact with nature could also be promoted during class time by taking students outside during lessons or by motivating them to engage in outdoor adventure experiences (Barton et al., 2016). It is also pertinent to provide opportunities for adults to freely engage with nature (Thompson et al., 2008).

## Conclusion

The current study advances previous research on the relation between contact with nature and pro-environmentalism. We demonstrate that the effect of adults’ current recreational contact with nature on their self-reported PEBs is partially explained by connectedness to nature. Additionally, we found that positive contact with nature during childhood improves adults’ self-reported PEBs in two ways: first, by explaining adults’ current experiences in nature and second, through the indirect effects of childhood experiences in nature on self-reported PEBs via current experiences in nature. To the best of our knowledge, our study is the first of this kind conducted in a developing country. Our findings are in line with previous studies (Pensini et al., 2016; Otto and Pensini, 2017; Evans et al., 2018), suggesting that the relations between positive contact with nature and pro-environmentalism found in developed countries can be extended to Brazil. Childhood experiences in nature seem to have a lasting effect until adulthood, encouraging nature experiences later in life, which, in turn, promote pro-environmentalism. Future cross-cultural studies that include participants from different developed (e.g., United States and Europe) and developing (e.g., Latin America, Asia, and Africa) countries will allow us to ensure our findings can be generalized across different cultures.

## Ethics Statement

This study was carried out in accordance with the recommendations of RESOLUÇÃO No. 510 DE 07 DEABRIL DE 2016 of the Conselho Nacional de Saúde. The protocol was approved by the Committee of Ethics in Research with Human Beings of the Universidade Estadual de Santa Cruz (No. 2.055.100). All subjects gave written informed consent in accordance with the Declaration of Helsinki.

## Author Contributions

CR and CP conceived and designed the study. CR analyzed the data and wrote an initial draft based on the results. SC critically revised the draft manuscript and made important changes in content.

## Conflict of Interest Statement

The authors declare that the research was conducted in the absence of any commercial or financial relationships that could be construed as a potential conflict of interest.
