# Supplementary material for: Nature Experiences and Adults’ Self-Reported Pro-environmental Behaviors: The Role of Connectedness to Nature and Childhood Nature Experiences
Source: Front Psychol. 2018 Jun 26;9:1055. doi: 10.3389/fpsyg.2018.01055 (PMC6036283; doi:10.3389/fpsyg.2018.01055)
Supplement: Supplementary file 2 [file Table_2.DOCX]

Table 2 (Supplementary)

Item’s loading from the structure matrix of the exploratory factor analysis of the Pro-Environmental Behavior scale.

|  | Factor Loadings | | |
| --- | --- | --- | --- |
| Items | 1 | 2 | 3 |
| *Trabalhei com outras pessoas para resolver um problema ambiental.*  Worked with others to address an environmental problem or issue. | 0.75 |  | -0.65 |
| *Comprei produtos “amigos” do ambiente ou com eficiência energética.*  Bought environmentally friendly and/or energy efficient products. | 0.66 | 0.34 | -0.35 |
| *Conversei com as pessoas em minha comunidade sobre questões ambientais.*  Talked to others in my community about environmental issues. | 0.65 | 0.35 | -0.41 |
| *Reciclei papel, plástico ou metal.*  Recycled paper, plastic and metal. | 0.60 |  | -0.36 |
| *Economizei água ou energia em minha casa.*  Conserved water or energy in my home. | 0.39 |  |  |
| *Assinei uma petição/abaixo-assinado acerca de uma questão ambiental.*  Signed a petition about an environmental issue. | 0.41 | 0.96 | -0.32 |
| *Votei em apoio a uma política pública/regulamentação que afeta o ambiente.*  Voted to support a policy/regulation that affects the local environment. | 0.35 | 0.67 |  |
| *Escrevi uma carta em resposta a uma questão ambiental.*  Wrote a letter in response to an environmental issue. | 0.37 | 0.33 | -0.76 |
| *Doei dinheiro para apoiar a proteção ambiental.*  Donated money to support local environmental protection. | 0.52 | 0.33 | -0.68 |
| *Participei como um membro ativo em um grupo ambiental*  Participated as an active member in a local environmental group. | 0.51 |  | -0.51 |

*Note.* Correlations < 0.32 were omitted.
